# Multiple host switching events shape the evolution of symbiotic palaemonid shrimps (Crustacea: Decapoda)

**DOI:** 10.1038/srep26486

**Published:** 2016-06-01

**Authors:** Ivona Horká, Sammy De Grave, Charles H. J. M. Fransen, Adam Petrusek, Zdeněk Ďuriš

**Affiliations:** 1 University of Ostrava, Faculty of Science, Department of Biology and Ecology, and Institute of Environmental Technologies, Chittussiho 10, Ostrava, CZ-710 00, Czech Republic; 2 Charles University in Prague, Faculty of Science, Department of Ecology, Viničná 7, Prague, CZ-12844, Czech Republic; 3 Oxford University Museum of Natural History, Parks Road, Oxford, OX1 3PW, United Kingdom; 4 Department of Marine Zoology, Naturalis Biodiversity Center, P.O. Box 9517, 2300 RA Leiden, The Netherlands

## Abstract

The majority of the almost 1,000 species of Palaemonidae, the most speciose family of caridean shrimp, largely live in symbioses with marine invertebrates of different phyla. These associations range from weak epibiosis to obligatory endosymbiosis and from restricted commensalism to semi-parasitism, with the specialisation to particular hosts likely playing a role in the diversification of this shrimp group. Our study elucidates the evolutionary history of symbiotic palaemonids based on a phylogenetic analysis of 87 species belonging to 43 genera from the Indo-West Pacific and the Atlantic using two nuclear and two mitochondrial markers. A complementary three-marker analysis including taxa from GenBank raises this number to 107 species from 48 genera. Seven larger clades were recovered in the molecular phylogeny; the basal-most one includes mostly free-living shrimp, albeit with a few symbiotic species. Ancestral state reconstruction revealed that free-living forms likely colonised cnidarian hosts initially, and switching between different host phyla occurred multiple times in palaemonid evolutionary history. In some cases this was likely facilitated by the availability of analogous microhabitats in unrelated but morphologically similar host groups. Host switching and adaptations to newly colonised host groups must have played an important role in the evolution of this diverse shrimp group.

Organisms do not live in isolation, but in close relation with an assemblage of phylogenetically close or remote species, often establishing symbioses. Symbiosis commonly refers to associations of two or more organisms of different taxa (often evolutionarily widely separated) that may last for the lifetime of one or all partners^1^ but also includes looser and/or shorter temporal associations^2^. Some authors, however, define symbiosis in a more restricted concept as an intimate interaction between different organisms in which at least one of the parties is obligatorily dependent on the association for a part of its life history^3^ (thus including parasitism, mutualism, and phoresis).

A significant portion of the high diversity of larger bodied crustaceans on coral reefs is formed by caridean shrimps (infraorder Caridea), many of which take part in symbiotic relationships, particularly in the family Palaemonidae, but also in Alpheidae and Hippolytidae. Together with alpheids, the prominent systematic group of shrimps participating in these relationships (an estimated 60–80%^4^) are in the family Palaemonidae, for which numerous host–symbiont associations are known^5^. Palaemonid shrimps with around 1,000 described species, combining the polyphyletic subfamilies Pontoniinae and Palaemoninae, and recently shown to also include the families Gnathophyllidae and Hymenoceridae^6^, comprise the largest family of the whole infraorder^7^. Representatives of the family primarily occur in tropical and subtropical latitudes in shallow-water benthic habitats, with their highest marine diversity occurring in the Indo-West Pacific area (IWP).

Free-living marine palaemonid species are usually larger and more slender and are typically considered micro-browsers or scavengers. By contrast, symbiotic palaemonid shrimps are generally small-bodied with a cryptic marine lifestyle. These shrimps occur symbiotically with a large variety of cnidarians, but equally with a variety of other host organisms, such as sponges, molluscs, echinoderms, ascidians (Fig. 1)^8^, and even other decapod crustaceans^9^. Their associations range from weak epibiosis to obligatory endosymbiosis and from restricted commensalism to semi-parasitism^10^,^11^, although the details of the majority of these interactions remain unclear. This symbiotic mode of life is accompanied by a vast array of morphological *bauplan* changes, such as a reduction of spines and protrusions, as well as extensive modifications to all pereiopods, mouthparts, and even their eyes^5^,^12^.

Together with coadaptation and cospeciation, host switching has been recognised as one of the main drivers of speciation in many coevolutionary studies^13^ (although actual evidence of cospeciation might be relatively rare^14^). Several studies have demonstrated that host associations are generally conserved across phylogenies and that switches between distantly related hosts are infrequent^15^,^16^. Such a conserved trait is logical as morphological, behavioural and physiological specialisations allow a symbiont to efficiently exploit a single host, but makes it difficult to exploit new, phylogenetically distant hosts. However, symbiotic lineages do occasionally switch to new and even distantly related host species^17^.

Host switching has been widely studied in terrestrial ecosystems, most notably in herbivorous insects and parasites, but it remains largely unstudied in marine environments^17^. An illustrative series of host-symbiont interactions from different geographic regions of the world’s oceans was described by Sotka^18^, who provided examples of local adaptations in host use among such phylogenetically distant marine taxa of invertebrates as a sessile polychaete, gastropods, and amphipods. Reports of host switching have primarily come at within-phylum level, e.g. myzostomids associated with crinoid echinoderms^19^ and trematode parasites in snails^20^. Switching of invertebrate symbionts between different host phyla, however, has been reported by Goto *et al.*^17^ for galeommatoidean bivalves, symbionts of crustaceans, echiurans, sipunculans, and holothurians. Recently, Kou *et al.*^21^ identified divergent evolutionary pathways in symbiotic palaemonid shrimps (as ‘subfamily Pontoniinae’ – *sensu* De Grave & Fransen, 2011^7^) based on a preliminary analysis of 26 species (all IWP) in 23 genera, representing less than 5% of the total diversity of the taxon.

Here, we present results from a complementary but significantly extended phylogenetic analysis of palaemonid shrimp symbioses with hosts of different phyla. The dataset comprises of 87 species from 43 genera in a four-marker analysis (based on sequences of two nuclear genes, for 18S rRNA and histone 3 (H3), and of two mitochondrial genes, for cytochrome *c* oxidase subunit I (COI) and 16S rRNA), and 107 species from 48 genera in a three-marker analysis (based on H3, 16S and COI sequences), representing nearly 20% of known biodiversity at the species level and 40% of all known genera, and a much wider host association spectrum than analysed before. A comparable dataset of palaemonid shrimps, based on 101 IWP species from 42 genera, was recently analysed by Gan *et al.*^22^. Our analyses nevertheless differ significantly from this work by the inclusion of Atlantic taxa, particularly from the western Atlantic (WA), which is the second centre of speciation for the group, as well as the ancestral state reconstruction of host associations.

For the purpose of this contribution, we consider a palaemonid shrimp species to be ‘free-living’ if in general they do not live on or inside a host animal, although they can potentially enter into infrequent short-term relationships with a putative host taxon, for example residing for a limited time in a single coral colony. Symbiotic species (i.e. not free-living) are those which are regularly recorded on the surface (ectosymbiosis) or inside body cavities (endosymbiosis) of animal hosts, although they occasionally may occur away from these, for example when searching for mates. Symbionts were assigned to a host phylum on the basis of the extensive body of taxonomic literature for this group, with specific hosts for all voucher specimens given in Supplementary Table S1. The term ‘host switching’ is herein specifically applied to a change of host across different phyla.

## Results and Discussion

### Molecular phylogenetic analysis

The phylogenetic tree based on the combined dataset for four markers (87 species from 43 genera) is basally divided into two supported clades, Clade 1 and combined Clades 2–7 (Fig. 2). Within the second clade, five major clades are recovered, all of which are highly supported (Clades 2–6; with Bayesian posterior probabilities [BI] 0.96–1.00), and a further weaker supported Clade 7 (BI 0.93), which comprises multiple lineages with unresolved intra-clade phylogenetic relations.

The phylogenetic tree based on the combined dataset for three (H3, 16S and COI) markers (107 species from 48 genera; Fig. 3, Supplementary Fig. S1) includes a further 20 species, not used in the main four-marker analysis. Those species belong to 12 genera from which five, i.e. *Bathymenes*, *Dactylonia*, *Echinopericlimenes*, *Philarius*, and *Pontonia*, are not included in the four-marker analysis; additional representatives of the genera *Palaemonella*, *Cuapetes*, *Laomenes*, *Phycomenes*, *Harpiliopsis* and *Periclimenaeus* are nested in clades with their congeneric species.

**Figure 1:**
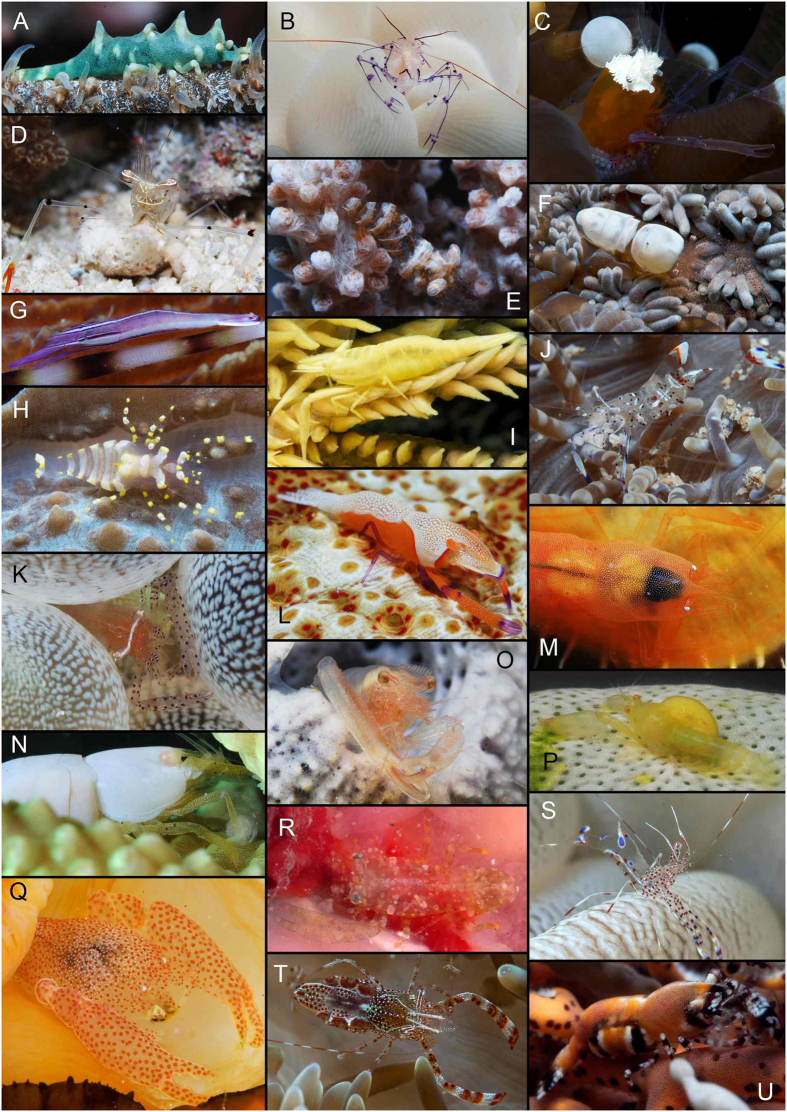
Examples of symbiotic shrimps from the main palaemonid clades (as in Figs 2, 3, 4), with one symbiotic pandalid species (**A**) as the outgroup. (**A**) *Miropandalus hardingi* on the black coral *Cirrhipathes* sp. (**B**) *Vir philippinensis* (clade 1) on the stony coral *Plerogyra sinuosa*. (**C**) *Cuapetes kororensis* (clade 1) on the stony coral *Heliofungia actiniformis*. (**D**) Free-living *Cuapetes tenuipes* (clade 1). (**E**) *Hamodactylus noumeae* (clade 2) on the soft coral *Nephthea* sp. (**F**) *Hamopontonia corallicola* (clade 2) on the stony coral *Goniopora* sp. (**G**) *Stegopontonia commensalis* (clade 2) on the sea urchin *Echinotrix calamaris*. (**H**) *Pliopontonia furtiva* (clade 3) on the corallimorpharian *Amplexidiscus fenestrafer*. (**I**) *Laomenes amboinensis* (clade 3) on a crinoid. (**J**) *Ancylomenes holthuisi* (clade 3) on the sea anemone *Heteractis aurora*. (**K**) *Periclimenes ornatus* (clade 5) on the sea anemone *Entacmaea quadricolor*. (**L**) *Zenopontonia rex* (clade 5) on the holothurian *Thelenota anax*. (**M**) *Anchistus custoides* (clade 5) in the pen shell *Atrina vexillium*. (**N**) *Coralliocaris superba* (clade 5) on the stony coral *Acropora* sp. (**O**) *Thaumastocaris streptopus* (clade 5) in a tube sponge. (**P**) *Periclimenaeus storchi* (clade 5) in the ascidian *Didemnum molle*. (**Q**) *Conchodytes meleagrinae* (clade 6) in the pearl oyster *Pinctada margaritifera*. (**R**) *Ascidonia quasipusilla* (clade 6) in an ascidian. (**S**) *Periclimenes yucatanicus* (clade 7) on the sea anemone *Condylactis gigantea*. (**T**) *Periclimenes rathbunae* (clade 7) on the sea anemone *Stichodactyla helianthus*. (**U**) *Periclimenes perryae* (clade 7) on the basketstar *Astrophyton muricatum*. All photos by CHJMF.

Clade 1 contains predominantly IWP free-living taxa of the genera *Cuapetes* (Fig. 1C,D), *Exoclimenella*, *Palaemonella*, *Periclimenella*, and coral-associated taxa of the genera *Harpilius*, *Ischnopontonia* and *Vir* (Fig. 1B). Based on the three-marker analysis (Fig. 3), the cnidarian-associated species of the genus *Philarius*, as well as crinoid-associated *Palaemonella pottsi* and free-living *Cuapetes elegans*, also belong to this clade. In contrast, Clades 2–7 largely comprise symbiotic palaemonid shrimps, with only a few free-living species: *Ancylomenes kuboi* and *Phycomenes sulcatus* from Clade 3; and *Gnathophyllum americanum* and *Hymenocera picta* that form together Clade 4.

Clade 2 contains a polytomy of four lineages, i.e. the IWP cnidarian-associated genera *Hamodactylus* (Fig. 1E) with *Hamopontonia* (Fig. 1F); *Manipontonia*; *Rapipontonia* with the crinoid-associated *Brucecaris*; and a subclade of other echinoderm-associated genera living on crinoids (*Pontoniopsis*) and on sea urchins (*Tuleariocaris* and *Stegopontonia*; Fig. 1G).

Clade 3 mainly consists of cnidarian-associated species of the genera *Ancylomenes*, *Pliopontonia* (Fig. 1H), *Periclimenes* (which, being polyphyletic, is scattered across multiple clades), and *Phycomenes* (Fig. 1J) containing also two above-mentioned free-living species, *A. kuboi* and *P. sulcatus*. A compact genetic lineage of echinoderm-associated species of the genera *Araiopontonia*, *Laomenes* (Fig. 1I), *Unguicaris*, and *Periclimenes* (*P. commensalis*, *P. cristimanus*, and *P. zanzibaricus*, and, as indicated by three-marker analysis, also *P. affinis*) is nested within the above-mentioned predominantly cnidarian-symbiont clade. All analysed representatives here belong to IWP taxa.

Clade 4 is composed of only two free-living ‘gnathophylloid’^6^ species of the genera *Gnathophyllum* and *Hymenocera*, both collected in the IWP, although *G. americanum* as well as some other species of the genus are also found in the Atlantic.

Clade 5 contains the IWP mollusc-associated genera *Anchistus* (Fig. 1M) and *Paranchistus* together with *Dasella* (living in solitary ascidians), an assemblage of echinoderm-associated taxa including, in addition to representatives of the genera *Lipkemenes* and *Zenopontonia* (Fig. 1L), also *Periclimenes colemani*, as well as diverse *Periclimenes* species ectosymbiotic on sea anemones (*P. inornatus* and *P. ornatus*; Fig. 1K) or corals (*P. kempi*). A second subclade within Clade 5 contains the IWP scleractinian coral-associated genera *Coralliocaris* (Fig. 1N), *Harpiliopsis*, and *Platycaris*. The third subclade comprises the obligatory sponge endosymbionts of the genera *Nippontonia*, *Orthopontonia*, *Thaumastocaris* (Fig. 1O), *Typton*, and *Periclimenaeus*, as well as one species of the latter genus inhabiting compound ascidians. An additional ascidian-endosymbiotic *Periclimenaeus* is recovered in this clade by the three-marker analysis (Fig. 3).

Clade 6 comprises mollusc-endosymbiotic representatives of the genera *Anchiopontonia* and *Conchodytes* (IWP, Fig. 1Q) and ascidian-endosymbiotic representatives of the genera *Ascidonia* (WA, Fig. 1R) and *Odontonia* (IWP). Additional species of the genera *Pontonia* (bivalve associates, Atlantic) and *Dactylonia* (ascidian associates, IWP) belong to this clade according to the three-marker analysis (Fig. 3).

**Figure 2:**
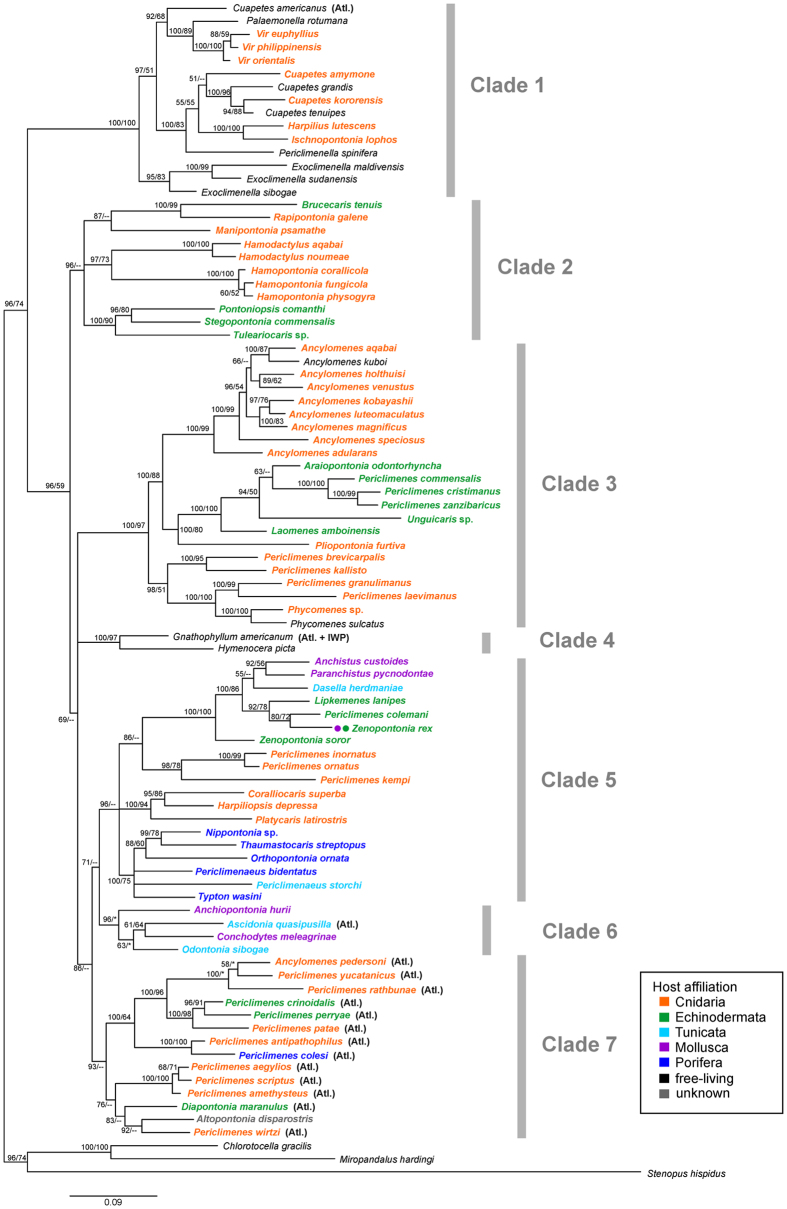
Phylogenetic tree of symbiotic Palaemonidae shrimp taxa resolved by Bayesian inference based on the combined dataset for four genes (COI, 16S, H3, 18S). Bayesian posterior probabilities and RAxML bootstrap support expressed as percentages are indicated. Dash (–) indicates RAxML values <50; asterisk (*) indicates different topology of RAxML tree. Major clades and host affiliations are highlighted. Atlantic taxa are indicated (Atl.), other species are from the Indo-West Pacific area.

**Figure 3:**
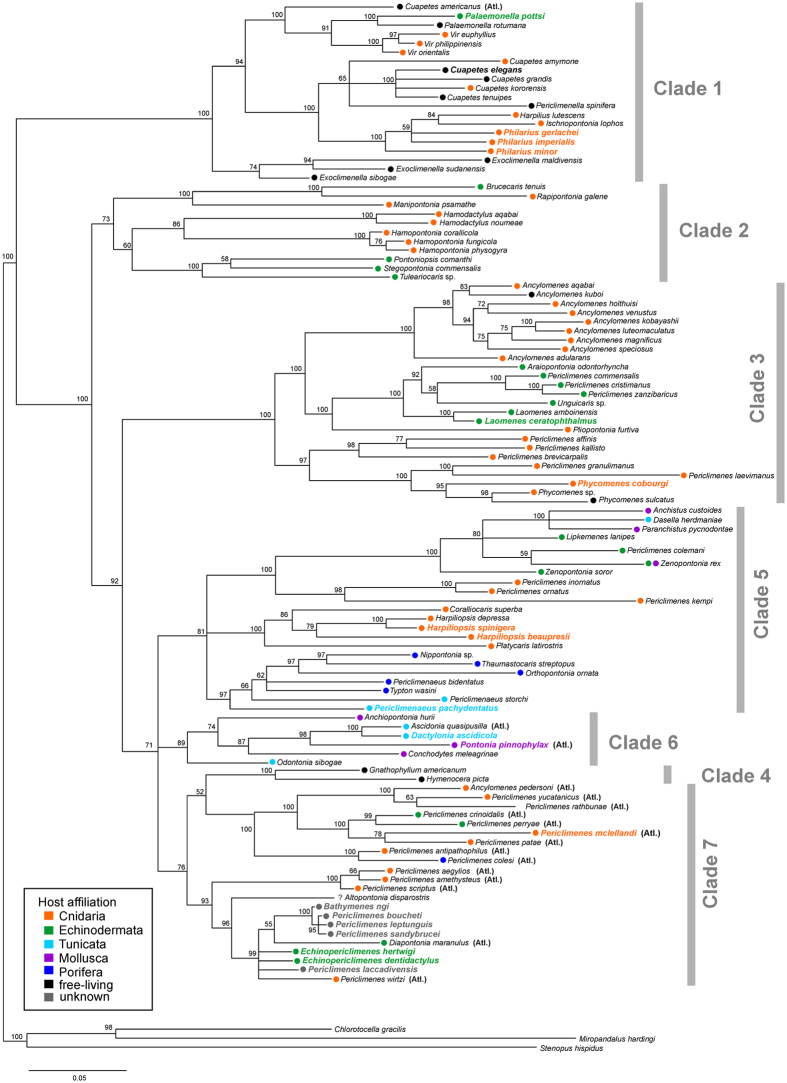
Phylogenetic tree of symbiotic Palaemonidae shrimp taxa (including sequences of taxa retrieved from GenBank) resolved by Bayesian inference based on the combined dataset for three genes (COI, 16S, H3). Bayesian posterior probabilities expressed as percentages are indicated (for ML tree topology, see Supplementary Fig. S1). Major clades and host affiliations are highlighted. Names of newly added taxa are in colour. Atlantic taxa are indicated (Atl.), other species are from the Indo-West Pacific area.

Clade 7 is basally only marginally supported (BI 0.93). It contains a series of distinct lineages, here provisionally drawn together in one combined branch. However, both the four-and three-marker analyses indicate dominance of Atlantic taxa in this clade (Figs 2 and 3), which might indicate a common origin. The placement of additional Atlantic taxa in the phylogeny (also in Clades 1, 4, and 6) is strongly suggestive of multiple invasions of palaemonid shrimps into the proto-Atlantic from the Pacific. Clade 7 represents assemblages of diverse host and depth affiliations. A basally well supported subclade covers the western Atlantic anemone-associated *Ancylomenes* and *Periclimenes* species (*P. yucatanicus*, *P. rathbunae*; Fig. 1S,T), together with a series of other *Periclimenes* species associated with antipatharian corals (*P. antipathophilus* and *P. patae*; also *P. mclellandi* in Fig. 3) and with echinoderms (*P. crinoidalis*, *P. perryae*) or sponges (*P. colesi*). The remaining subclade represents a basally unresolved assemblage of the following lineages: the eastern Atlantic species of anemone-associated *Periclimenes* (containing *P. amethysteus*, the type species of the genus), and a lineage with two deep-water taxa, the IWP *Altopontonia disparostris* (with unknown host affiliation) and the Atlantic *Diapontonia maranulus* (sea-urchin associate), together with the eastern Atlantic antipatharian-associated *P. wirtzi*. The three-marker analysis (Fig. 3) markedly widens this assemblage to include a series of IWP deep-water *Periclimenes*, in addition to two other IWP deep-water genera *Echinopericlimenes* (echinoid-associated) and *Bathymenes* (cnidarian or echinoid-associated, but mostly without known host affiliation).

Our phylogenetic analyses recover several genera as polyphyletic or paraphyletic, e.g. *Ancylomenes*, *Cuapetes*, *Periclimenes*, and *Zenopontonia*. The genus level systematics of Palaemonidae clearly requires further scrutiny, which is beyond the scope of the present study. To resolve the internal composition of these non-monophyletic taxa, extended molecular datasets combined with morphological analyses, and studies incorporating taxa from all main tropical and subtropical regions, are needed. For example, the long recognised polyphyletic genus *Periclimenes*^22^,^23^,^24^ can only be resolved if 1) an unambiguous position in a well-supported clade of the type species is recovered for the eastern Atlantic *P. amethysteus*, 2) the relationship with presently congeneric eastern and western Atlantic taxa is resolved, and 3) many more IWP taxa currently still included in this “waste basket” genus and related taxa are included in a thorough phylogenetic framework. In the present study, we thus adhere to the current systematic composition of *Periclimenes* species, as well as other polyphyletic/paraphyletic genera^7^.

### Parallel evolution of host associations

**Figure 4:**
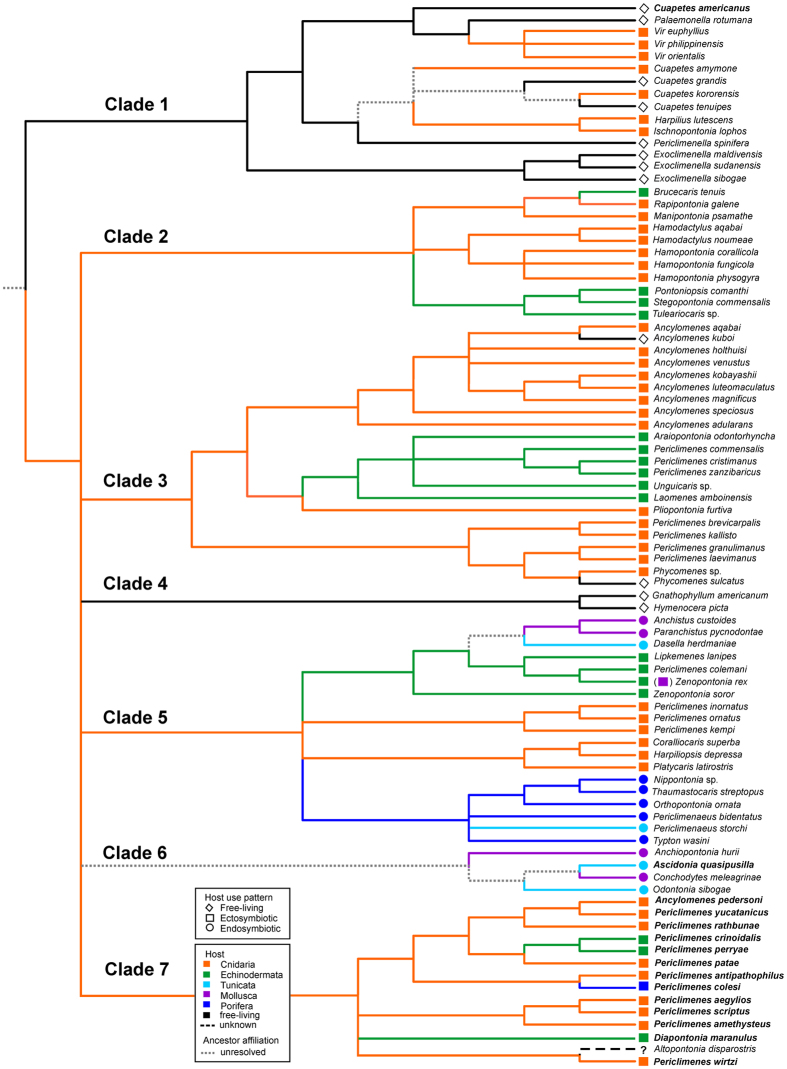
Ancestral state reconstruction of host associations of palaemonid shrimps, based on the topology of Bayesian inference tree from the four-marker analysis (shown in Fig. 2), assessed by the parsimony criterion. Branches supported by <90% Bayesian posterior probability together with <70% bootstrap support are collapsed. The colour and shape of each symbol indicate the host taxon and host-use pattern, respectively. Atlantic species are marked in bold; other species were collected in the Indo-West Pacific area.

To investigate the evolutionary pathways of symbiotic lifestyles, information on the lifestyle (symbiotic vs. free-living), host taxon and host association mode (endo- vs. ectosymbiotic), was mapped onto the phylogenetic four-gene tree and presented in Fig. 4.

Symbiotic associations with cnidarians, echinoderms, tunicates, bivalves and sponges occur multiple times in the present phylogeny, indicative of multiple host invasions in the evolution of the clade as a whole.

Free-living taxa are largely concentrated in Clade 1 (Figs 2, 3, 4). These shrimps are characterised by an elongate body shape with a dentate rostrum and slender, long, symmetrical second chelipeds. In addition to these species, some obligatory symbionts are also present in this clade, such as those from the genus *Cuapetes*, which have a similar *bauplan* to these free-living genera. It should be noted that the latter genus contains several species living with scleractinian (Fig. 1C), alcyonarian or antipatharian hosts^23^,^25^,^26^. However, a morphologically very disparate taxon, *Ischnopontonia lophos*, is also part of this clade. This species inhabits the network of narrow spaces amongst the cylindrical corallites, fully hidden under densely set tentacles of coral polyps in the genus *Galaxea*. It shares this habitat with another palaemonid, *Anapontonia denticauda*, and the alpheid *Racilius compressus.* Remarkably, all these species have similar tail fan structures and laterally compressed bodies^5^,^27^. Most symbiotic shrimps within this clade of primarily free-living forms are associated almost exclusively with cnidarian hosts. At the species level, the only exception is *Palaemonella pottsi*, an obligatory associate of crinoids^28^,^29^ (Fig. 3).

Free-living taxa are rare in the remaining clades. A particularly notable exception is *Ancylomenes kuboi* (Clade 3). Species of this genus are predominantly anemone-associated fish-cleaners. Some species, however, seem to have undergone an evolutionary reversal to a free-living mode of life. *Ancylomenes kuboi*, as well as *A. tosaensis* (not included in our analysis), live in close vicinity to sea anemones but do not appear to be obligate associates. Both species possess simple dactyli on the ambulatory pereiopods, typical for free-living species (e.g. in Clade 1), while their anemone associated congeners have bifurcated dactyli^24^. Similarly, *Gnathophyllum americanum* and *Hymenocera picta* (Clade 4) can also be formally considered as free-living, although always remaining in relative proximity to echinoderms, which is likely related to their feeding biology.

Below, we provide more details of the host-symbiont relationships for the various groups of shrimps which independently established such relationships, and showcase the extraordinary breath of these associations.

#### Cnidarians

One of the most distinctive (morphologically as well as behaviourally) cnidarian-associated genera is *Ancylomenes* (Clade 3). Of the 22 known species, the majority live in association with sea-anemones^24^,^30^. For six of these species, fish-cleaning behaviour has been directly observed (e.g.^24^,^31^). Some species also occasionally live on other cnidarians (e.g. stony corals of the genera *Heliofungia* and *Euphyllia*, jellyfish of the genus *Phyllodiscus* or the corallimorpharian *Amplexidiscus*; IH, CHJMF, ZĎ – pers. observ.), and some *Ancylomenes* species are substrate dwellers (e.g. *A. kuboi*; IH, ZĎ – pers. observ.), illustrating an example of intrageneric evolutionary and ecological plasticity within symbiotic shrimps. However, the genus *Ancylomenes* appears as polyphyletic in the present analysis, due to the separate evolutionary position of its Atlantic member *A. pedersoni* (Clade 7), an example of remarkable morphological, ecological, and behavioural parallelism among anemone-associated shrimps^24^,^31^.

The ‘disk-anemone’-associated shrimp *Pliopontonia furtiva* (Fig. 1H, Clade 3) blankets its body with the host’s soft tissues (IH, ZĎ, CHJMF – pers. observ.), potentially precursor behaviour to an endosymbiotic mode of life. In the present analyses, however, it shows a closer relation to echinoderm ectosymbiotic shrimps (Figs 2, 3, 4).

#### Echinoderms

Notably, all echinoderm-associated clades of palaemonids (Clades 1–3, 5, 7; Figs 2, 3, 4) are represented by exclusively ectosymbiotic forms, although endosymbiotic forms are known in other groups of Decapoda, e.g. crabs in the cloaca of holothurians^32^. Interestingly, echinoderm associates are observed among deep-water forms of the genera *Periclimenes*, *Echinopericlimenes* and *Bathymenes* (all in Clade 7; see Fig. 3), as well as among shallow-water ‘gnathophylloid’ shrimps; most IWP as well as Atlantic species of the latter group live with sea urchins^33^,^34^ but free-living forms are also represented in these taxa (Clade 4). Similarly, the IWP ‘hymenoceroid’ shrimps from the same clade, here also regarded as free-living, exhibit an affinity to an echinoderm association; for example, the harlequin shrimp *Hymenocera picta* is a predator of sea stars^35^. *Zenopontonia rex*, still widely known as *Periclimenes imperator* (Fig. 1L, Clade 5) seems to be a rare case of possible ontogenetic host switching, living on sea cucumbers and, occasionally, on sea stars (echinoderms) as juveniles, and on nudibranch sea slugs (molluscs) as adults^36^,^37^.

An elongated body with asymmetrical chelipeds and stout walking legs are features typical for the echinoid-associated genera *Stegopontonia* and *Tuleariocaris* (Clade 2), similar in morphology to the representatives of the genus *Periclimenes* from Clade 3 (*P. cristimanus*, *P. zanzibaricus*), also symbionts on sea urchins^5^,^28^.

Another *Periclimenes* from echinoids, *P. colemani*, belongs to the lineage containing *Zenopontonia* and *Lipkemenes* (Clade 5) with the same echinoderm host affiliation^36^,^37^. These species show a general morphological resemblance to the ascidian- or bivalve-associated genera *Dasella*, *Anchistus* and *Paranchistus*, with a smooth body and down-curved rostrum with minute or reduced dentition^5^.

#### Sponges

Sponge ectosymbionts are rare among palaemonid shrimps; notable examples are *Periclimenes incertus*^38^, the deep-sea *P. forcipulatus*^39^, and the western Atlantic *P. colesi*^40^ (Clade 7). The last-mentioned species belongs to the western Atlantic *Periclimenes iridescens* species complex which are generally shallow-water, predominantly cnidarian-associated shrimps (occurring with gorgonarians and antipatharians)^40^. This species is thus another example of intra-generic host switching.

By contrast, endosymbiotic associations with sponges (Clade 5) are ubiquitous, notably in the genera *Nippontonia*, *Orthopontonia*, *Periclimenaeus*, *Thaumastocaris* (Fig. 1O) and *Typton*. In fact, after cnidarians, sponges are the most frequently inhabited hosts for symbiotic palaemonid shrimps and, in particular, are the most frequent hosts for endosymbiotic shrimps^8^,^11^. The endosymbiotic life in sponges is quite demanding for shrimps regarding their *bauplan* adaptations for life inside narrow tubes^11^, and available food sources. While bivalve- or ascidian-associated shrimps feed mainly on mucus produced by their hosts and food particles collected in it, most sponge-inhabiting palaemonids have developed the ability to feed on sponge tissues, as demonstrated for shrimps of the genera *Typton*, *Periclimenaeus*, *Onycocaris* and *Thaumastocaris*; however, these shrimps may provide reciprocal services to their host, perhaps of a mutualistic nature^11^. The present paraphyletic composition of the endo-spongobiotic shrimps suggests their common origin.

#### Ascidians

The taxa included in the present analyses indicate that multiple invasions of palaemonids into ascidians have occurred. The pantropical genus *Periclimenaeus* associated with sponges or ascidians is nested within a clade of sponge-endosymbiotic shrimps (Clade 5). It actually consists of approximately 80 species. Looking at all species from that genus for which the host is known^5^,^8^, the ascidian-associated species represent about one third of them.

Ascidian-associated taxa occupy two morphologically different ascidian groups with distinctive host morphologies. Species of the genera *Ascidonia*, *Dasella*, *Dactylonia* and *Odontonia* (Clade 6) live inside solitary sea squirts with relatively large bodies, moving and feeding inside their branchial chamber. By contrast, some *Periclimenaeus* species (Fig. 1P, Clade 5; and possibly also *Colemonia*) occupy communal exhalatory channels of compound (colonial) ascidians^8^,^41^. Such a network of narrow subdermal channels is structurally similar to the internal channels of sponges, which host the majority of species of the genus *Periclimenaeus*^11^.

A further ascidian symbiont, *Dasella herdmaniae* (WA; Clade 5), displays, as noted above, a closer affinity to a subclade comprising bivalve endosymbionts *Anchistus* and *Paranchistus*, while the above group of ascidian symbionts (*Ascidonia*, *Dactylonia* and *Odontonia*) are closer to bivalve-associated *Anchiopontonia*, *Conchodytes* and *Pontonia* (Clade 6, combined from both analyses, see Figs 2, 3, 4). It is thus evident that the ascidian endosymbiotic species belong to at least three distant phylogenetic lineages, two of which show a closer relation to bivalve-mollusc endosymbionts while the third is related to sponge-inhabiting shrimps. The relation to bivalve symbionts of the first two lineages is supported also by their phenotypically similar smooth subcylindrical bodies with strong chelae and reduced dentition of body and rostrum^5^,^41^.

#### Molluscs

Our analysis uncovers evidence of at least two bivalve endosymbiotic host invasions (Clades 5, 6), one comprising the genera *Anchistus* and *Paranchistus*, and the second the genera *Anchiopontonia* and *Conchodytes* (Fig. 1Q), together with the Atlantic *Pontonia* (Figs 2 and 3). While the former is linked to an echinoderm-associated clade^42^, the ancestral host affiliation of the latter assemblage still remains unresolved (Fig. 4).

### Evolution of host switching

Some insights into the evolution of palaemonid symbioses has recently been provided by Kou *et al.*^21^ based on a very limited dataset, and Gan *et al.*^22^, utilising a number of taxa comparable to our analysis, but restricted to IWP species. Our results of the Ancestral State Reconstruction analysis (Fig. 4) cover a more extensive set of species, including a series of Atlantic taxa.

As De Grave *et al.*^6^ indicated, a lineage of the predominantly free-living forms (here Clade 1) might be a separate group within palaemonoids, not closely related to the present symbiotic shrimps of the combined Clade 2–7, and is to be considered separately. The ancestral life mode in Clade 1 is revealed here to be free-living, from which symbiotic forms evolved via multiple switches into cnidarian or crinoid ectosymbionts. A remarkably high ratio of symbiotic forms within this clade is due to underrepresentation of the free-living species from the most speciose genera *Cuapetes* and *Palaemonella*. A reversal back to the free-living habit is not revealed in Clade 1, and is generally rare among symbiotic shrimps (but see Clade 3). The study by Kou *et al.*^21^ included only a single free-living species, while the results of Gan *et al.*^22^ mirror ours in revealing a clade of free-living and cnidarian-associated taxa, with a single crinoid symbiont.

Symbioses with cnidarians most likely represent the ancestral state of the host associations of symbiotic palaemonid shrimps, and this association is also the most numerous across all clades in our analyses of recent taxa. Except for Clade 1, most other cnidarian-associated lineages apparently evolved directly from an ancestral cnidarian host affiliation. Those associations thus developed without intermediate interphylum host switching into multiple lineages that retained an ancestral ectosymbiotic habit. Kou *et al.*^21^ and Gan *et al.*^22^ also indicate the multiple evolutionary lineages of cnidarian symbioses in the recent palaemonid shrimps.

Echinoderm symbioses evolved multiple times during the evolution of palaemonid symbioses (Figs 2, 3, 4), as suggested also by other studies^21^,^22^. Except for *Palaemonella pottsi* (Fig. 3, Clade 1) which may have switched to crinoids directly from a free-living habit, all those nested in Clades 2–3, 5 and 7 evolved from basal symbioses with cnidarians by interphylum host switching, but retaining an ectosymbiotic life mode. Furthermore, as noted above, the IWP ‘hymenoceroid’ shrimps (Clade 4) also exhibit an affinity to the echinoderm associations.

Sponge-associations also evolved multiple times within Palaemonidae, although previous studies indicated only a single sponge-associated lineage of the genus *Periclimenaeus* and related endosymbiotic genera^21^,^22^. These taxa, inhabiting internal cavities of sponges, apparently originated from cnidarian ectosymbionts by a single interphylum host switching event, complemented by embracing an endosymbiotic habit as a further evolutionary novelty in this lineage. The ectosymbiont of sponges *Periclimenes colesi* from the Atlantic (Clade 7) occupies an isolated evolutionary position among cnidarian symbionts, separated from the above endosymbiotic genera. Other sponge ectosymbionts from IWP that were not included in our analyses (*Periclimenes incertus*^38^ and deep-sea *P. forcipulatus*^39^) also seem unrelated to the above sponge endosymbionts.

Ascidian-associated endosymbiotic shrimps have evolved at least three times (Fig. 4), in contrast to previous studies, which resolved only 1–2 lineages^21^,^22^. While the compound-ascidian-inhabiting *Periclimenaeus* species fall among sponge-associated congeners, the other two evolutionary lineages of shrimps inhabiting solitary ascidians (Fig. 2, Clade 5) show a consistent relationship to bivalve-associated shrimps, although their mutual phylogenetic positions and descendant host relations still remain unresolved.

The two mollusc-associated endosymbiotic shrimps living inside bivalves, as well as the third lineage, the ectosymbiotic *Zenopontonia rex* occurring on gastropods or echinoderms, have already been mentioned (Figs 2, 3, 4).

The latter example well illustrates the ecological plasticity of shrimp species allowing them to adapt to different hosts. Such a plasticity, as well as intraphylum host variabilities, occasional associations, or ‘semi-endosymbiotic’ relations mentioned above, highlights one evolutionary instrument allowing initial looser symbiotic relations and, evolving further by host switches, leading to more-or-less fixed ecto- or endosymbioses.

## Conclusions

Host switching events and colonization of host organisms clearly played a major role in the evolution of symbiotic palaemonid shrimps, as such events frequently occur within most lineages, free-living as well as symbiotic ones. Reversal back to a free-living mode of life is a rare phenomenon, only occasionally observed among ectosymbiotic lineages. Inter-phylum host switching to Cnidaria, Echinodermata, Mollusca, Porifera and Tunicata occurred multiple times in the evolution of Palaemonidae, often in distantly related taxa. Host switching from ecto- to endosymbiotic modes of life also occurred multiple times, resulting in species-specific associations with bivalve molluscs, sponges and ascidians. It can thus be inferred that host switching has substantially contributed not only to speciation and radiation of the group as a whole, but also the emergence of new body plans and ecological adaptations, which contribute to the exceptionally high diversity of symbiotic palaemonids. The inclusion of Atlantic taxa hints at multiple historical invasions of palaemonid shrimps between the Pacific and the Atlantic. Our phylogenetic analyses clearly demonstrates polyphyly or paraphyly of several genera as presently defined (e.g. *Ancylomenes*, *Cuapetes*, *Periclimenes*, *Zenopontonia*), once again highlighting the need for extensive systematic revision in the group.

## Methods

### Sampling and DNA extraction, amplification and sequencing

A total of 107 palaemonid shrimp species belonging to 48 genera were included in this study (see Supplementary Tables S1 and S2). Of these, 90 species from across the main biogeographic areas (73 from IWP; 5 from East Atlantic; 12 from West Atlantic) belonging to 43 genera were newly sequenced. In addition to free-living taxa, shrimp species were selected to cover all main symbiotic associations, i.e. with Porifera, Cnidaria, Mollusca, Echinodermata and Tunicata, as well as to cover wider shrimp diversity within each of those associations. Where possible, we used approximately comparable number of 1–3 species per genus, with exceptions of selected genera where wider spectrum of hosts is known or which are already regarded as polyphyletic.

Details of analysed specimens, host information, sampling locations, voucher identification numbers and GenBank accession numbers are provided in Supplementary Table S1. Additional sequences from GenBank that were included in the analyses are listed in Supplementary Table S2.

Shrimp specimens were collected by scuba diving or other standard sampling methods in diverse regions, including Caribbean Sea, Mediterranean Sea, Red Sea, South China Sea, Bismarck Sea and Great Barrier Reef, and were preserved in 70% ethanol for identification. Pieces of tissues (abdominal muscle, posterior pleopods, or eggs) were preserved in 96–99% ethanol for molecular analyses. Voucher specimens were deposited in collections of the following institutions: Museum of Tropical Queensland, Townsville, Australia (MTQ); National Museum of Natural History, Paris, France (MNHN); National Taiwan Ocean University (NTOU), Naturalis Biodiversity Center, Leiden, the Netherlands (formerly Rijksmuseum van Natuurlijke Historie, RMNH); and University of Ostrava, Czech Republic (UO).

Total genomic DNA was extracted from analysed tissues using the DNeasy Blood & Tissue Isolation Kit (QIAGEN) according to the manufacturer’s instructions. Partial segments of four genes were amplified by PCR: mitochondrial genes for cytochrome *c* oxidase subunit I (COI, 658 bp) and 16S rRNA (16S, ca. 501 bp); and nuclear genes for histone 3 (H3, 293 bp) and 18S rRNA (18S, ca. 663 bp).

Polymerase chain reactions were performed in 25-μl volumes containing 2 μl of DNA template, 0.4 μM forward and reverse primers, 0.15 mM dNTPs, 0.7 units of *Taq* DNA polymerase, recombinant (Thermo Scientific), distilled water, 10× PCR buffer, and 2 mM MgCl_2_ (up to 4 mM for some species where amplification with lower concentrations failed).

The mitochondrial genes for COI and 16S rRNA were amplified using the universal primer pairs LCO 1490/HCO 2198^43^ and 16Sar/16Sbr^44^. The nuclear genes for H3 and 18S rRNA were amplified using the primer pairs H3F/H3R^45^ and 18Sa2.0/18S9r^46^. The thermal cycling profiles conformed to the following parameters: COI – 2.5 min at 94 °C for initial denaturation, followed by 40 cycles of 30 s at 90 °C, 1 min at 48 °C, and 1 min at 72 °C and a final extension step at 72 °C for 10 min; H3 – according to the protocol of Li *et al.*^47^; 16S and 18S – 2.5 min at 90 °C, followed by 10 cycles of 50 s at 92 °C, 30 s at 42–48 °C, and 1 min at 72 °C, 36 cycles of 30 s at 92 °C, 40 s at 42–48 °C, and 1 min at 72 °C and a final extension step at 72 °C for 3 min.

The PCR products were purified using the GenElute PCR Clean-up kit (Sigma-Aldrich). Sanger sequencing reactions were performed using an ABI3730XL DNA Sequencer by Macrogen, Inc. (Seoul and Amsterdam).

### Phylogenetic analyses

The forward and reverse sequences were combined and visually analysed in Chromas v2.4.1. Multiple sequence alignments were constructed using the MUSCLE^48^ algorithm with the default setting, incorporated within the software package MEGA v6^49^. Alignments of the protein-coding genes COI and H3 were translated to amino acids to check for possible frameshift mutations and stop codons, which would indicate the presence of pseudogenes.

The substitution saturation for COI and H3 genes was tested in DAMBE v5.3^50^ using the index proposed by Xia *et al.*^51^. The 3^rd^ codon position of COI gene was found saturated and thus excluded from the subsequent analyses.

Highly divergent blocks were examined in alignments for ribosomal genes 16S and 18S using Gblocks v0.91b^52^ with default parameters but allowing gap positions. Variable and parsimony-informative sites in each gene were identified with MEGA v6^49^ (Supplementary Table S3).

Multigene datasets were assembled using the concatenation software SequenceMatrix^53^. The final four-marker dataset comprising 1859 characters (H3 – 293 bp, COI – 436 bp, 16S – 467 bp, and 18S – 663 bp) covers the main diversity of symbiotic palaemonid taxa themselves. The supplementary three-marker dataset comprising 1207 characters (COI – 436 bp, 16S – 478 bp, H3 – 293 bp) includes sequences of additional palaemonid species for which only 16S and occasionally H3 sequences are available, and which are not, thus, covered by the main dataset.

The datasets were analysed using Bayesian inference (BI) and maximum likelihood (ML) to explore the strength of the phylogenetic signal under different optimality criteria. The best-fit models of nucleotide substitution and optimal partition schemes were selected with PartitionFinder^54^ under the Akaike Information Criterion using the concatenated datasets. Evolutionary models were assigned to each codon position separately for protein-coding genes.

Bayesian analyses were conducted via the on-line CIPRES Science Gateway v3.3^55^ with MrBayes v3.2.6^56^ on XSEDE. The tree topology and evolutionary model parameters were permuted using a Markov chain Monte Carlo method (MCMC). The MCMC was set for independent variability of parameters in individual coding and non-coding genes under the models selected by PartitionFinder^54^ (Supplementary Table S3). Two independent MCMC runs of four chains each were run until converging to a stationary distribution (standard deviation of split frequencies <0.01; 3 × 10^6^ and 5 × 10^6^ generations for four-gene and three-gene dataset, respectively). Trees were sampled each 500 generations, 25% of trees were discarded as burn-in, and the remaining ones were used to construct the 50% majority rule consensus tree and to estimate the Bayesian posterior probabilities.

Maximum Likelihood analysis with associated automatic bootstrap replicates under GTRGAMMA model was performed using RAxML via the on-line CIPRES^55^ with the RAxML-HPC BlackBox v8.2.6 tool^57^.

All trees were rooted by outgroup taxa from the families Stenopodidae (*Stenopus hispidus*) and Pandalidae (*Miropandalus hardingi*, *Chlorotocella gracilis*) and were displayed using the online application ITOL (Interactive Tree Of Life) v3.0^58^ and FigTree v1.4.2^59^.

Ancestral state character reconstruction to evaluate the evolution of host associations was assessed by parsimony criterion, based on the topology of the final BI tree from the four-marker analysis. The analysis was performed in Mesquite v3.04^60^.

## Additional Information

**How to cite this article**: Horká, I. *et al.* Multiple host switching events shape the evolution of symbiotic palaemonid shrimps (Crustacea: Decapoda). *Sci. Rep.* **6**, 26486; doi: 10.1038/srep26486 (2016).

## Supplementary Material

Supplementary Information
