# Supplementary material for: Multiple host switching events shape the evolution of symbiotic palaemonid shrimps (Crustacea: Decapoda)
Source: Sci Rep. 2016 Jun 1;6:26486. doi: 10.1038/srep26486 (PMC4887867; doi:10.1038/srep26486)
Supplement: Supplementary Information [file srep26486-s1.pdf]

SUPPLEMENTARY INFORMATION

to accompany

Multiple host switching events shape the evolution of symbiotic palaemonid shrimps (Crustacea: Decapoda)

Ivona Horká, Sammy De Grave, Charles H.J.M. Fransen, Adam Petrusek, Zdeněk Ďuriš

Supplementary Table S1: Details of the analysed specimens and freshly obtained DNA sequences used in the phylogenetic analyses. Used abbreviations and symbols: EA, East Atlantic; WA, West Atlantic; IWP, Indo-West Pacific; MNHN, National Museum of Natural History, France; MTQ, Museum of Tropical Queensland, Australia; NTOU, National Taiwan Ocean University; PNG, Papua New Guinea; RMNH, Naturalis Biodiversity Center, Leiden, the Netherlands; UO, University of Ostrava, Czech Republic; ULLZ, University of Louisiana at Lafayette Zoological Collection, U.S.A; and USVI, U.S. Virgin Island; * - specific host not identified.

Supplementary Table S2: Additional sequences obtained from GenBank and used in the present analyses.

Supplementary Table S3: Alignment length (saturation test for protein-coding genes and GBlock for rRNA genes was applied), character variation, parsimony-informative sites, sample size and evolutionary models used for the genes in this study. Models for protein-coding genes (H3 and COI) are shown for the 1st, 2nd or 3rd codon positions. Used abbreviations: VS – variable sites, PI – parsimony-informative sites, N – number of sequences obtained within this study (with number of sequences used from GenBank in parentheses).

Supplementary Figure S1: Phylogenetic tree of symbiotic Palaemonidae shrimp taxa (including sequences of taxa retrieved from GenBank) resolved by RAxML analysis based on the combined dataset for three genes (COI, 16S, H3). Bootstrap supports >50 are displayed.

Supplementary Table S1: Details of the analysed specimens and freshly obtained DNA sequences used in the phylogenetic analyses.

Analysed taxa	Region	Sampling location	Host	Voucher ID	GenBank accession numbers			
					H3	COI	16S	18S
**PALAEMONIDAE Rafinesque, 1815**								
Altopontonia Bruce, 1990								
A. disparostris Bruce, 1990	IWP	Norfolk Ridge	no data	RMNH.CRUS.D.51028	KU065026	KM921671	KU064797	N/A
Ancylomenes Okuno & Bruce, 2010								
A. adularans (Bruce, 2003)	IWP	Madang Bay, PNG	Cnidaria: Actiniaria*	MNHN IU-2013-11101	KU065027	KU064944	KU064798	KU064869
A. aqabai (Bruce, 2008)	IWP	Aqaba, Jordan	Cnidaria: Scyphozoa: *Cassiopea andromeda*	UO Aq09-26	KU065028	KU064945	KU064799	KU064870
A. holthuisi Bruce, 1969	IWP	Nhatrang Bay, Vietnam	Cnidaria: Actiniaria: *Stichodactyla haddoni*	UO V08-21B	KU065029	KU064946	KU064800	KU064871
A. kobayashii (Okuno & Nomura, 2002)	IWP	Okinawa, Japan	Cnidaria: Actiniaria*	NTOU M01865	KU065030	KU064947	KU064801	KU064872
A. kuboi Bruce, 2010	IWP	Nhatrang Bay, Vietnam	Cnidaria: Actiniaria: *Actinodendron* sp.	UO V08-120A	KU065031	KU064948	KU064802	KU064873
A. luteomaculatus Okuno & Bruce, 2010	IWP	Okinawa, Japan	Cnidaria: Actiniaria*	NTOU M01866	KU065033	KU064950	KU170693	KU170699
A. magnificus (Bruce, 1979)	IWP	Nhatrang Bay, Vietnam	Cnidaria: Ceriantharia*	UO V08-109	KU065034	KU064951	KU064804	KU064875
A. pedersoni (Chace, 1958)	WA	St. Thomas, USVI	Cnidaria: Actiniaria: *Bartholomea annulata*	UO USVI-5	KU065035	KU065003	KU064805	KU064876
A. speciosus (Okuno, 2004)	IWP	Lizard Island, Australia	Cnidaria: Actiniaria: *Heteractis* sp.	MTQ W-33108	KU065036	KU064952	KU170694	KU170698
A. venustus (Bruce, 1989)	IWP	Nhatrang Bay, Vietnam	Cnidaria: Actiniaria: *Megalactis* sp.	UO V08-79A	KU065037	KU064953	KU064806	KU064877
Anchiopontonia Bruce, 1992								
A. hurii (Holthuis, 1981)	IWP	Okinawa, Japan	Mollusca: Bivalvia*	NTOU M01830	KU065038	KU064955	N/A	N/A
Anchistus Borradaile, 1898								
A. custoides Bruce, 1977	IWP	Okinawa, Japan	Mollusca: Bivalvia*	NTOU M01867	KU065039	KU064954	KU064807	KU064878
Araiopontonia Fujino & Miyake, 1970								
A. odontorhyncha Fujino & Miyake, 1970	IWP	Okinawa, Japan	Echinodermata: Crinoidea*	NTOU M01868	KU065040	KU064956	N/A	N/A
Ascidonia Fransen, 2002								
A. quasipusilla (Chace, 1972)	WA	Curaçao, West Indies	Tunicata: Ascidiacea: *Pyura torpida*	RMNH.CRUS.D.57050	KU065041	KU064957	KU064808	N/A
Brucecaris Marin & Chan, 2007								
B. tenuis (Bruce, 1969)	IWP	Lizard Island, Australia	Echinodermata: Crinoidea*	MTQ W-33309	KU065042	KU064958	KU064809	KU064879
Conchodytes Peters, 1852								
C. meleagrinae Peters, 1852	IWP	Nhatrang Bay, Vietnam	Mollusca: Bivalvia: *Pinctada* sp.	UO V08-100	KU065043	KU064959	KU064810	KU064880
Coralliocaris Stimpson, 1860								
C. superba (Dana, 1852)	IWP	Aqaba, Jordan	Cnidaria: Scleractinia: *Acropora* sp.	UO Aq09-40	KU065044	KU064960	KU064811	KU064881
Cuapetes Bruce, 2004								
C. americanus (Kingsley, 1878)	WA	Curaçao, West Indies	Porifera: *Desmapsamma anchorata*	RMNH.CRUS.D.57051	KU065045	KU064961	N/A	N/A
C. amymone (De Man, 1902)	IWP	Lizard Island, Australia	Cnidaria: Scleractinia: *Acropora* sp.	MTQ W-33116	KU065046	KU064962	KU064812	KU064882
C. grandis (Stimpson, 1860)	IWP	Aqaba, Jordan	free-living	UO Aq09-58A	KU065047	KU064963	KU064813	KU064883
C. kororensis (Bruce, 1977)	IWP	Lizard Island, Australia	Cnidaria: Scleractinia: *Heliofungia actiniformis*	MTQ W-33107	KU065048	KU064964	N/A	KU064884
C. tenuipes (Borradaile, 1898)	IWP	Nhatrang Bay, Vietnam	Cnidaria: Actiniaria: *Actinodendron* sp.	UO V08-48	KU065049	KU064965	KU064814	KU064885
Dactylonia Fransen, 2002								
D. ascidicola (Borradaile, 1898)	IWP	Bali, Indonesia	Tunicata: Ascidiacea: *Ascidia* sp.	RMNH.CRUS.D.48678	N/A	N/A	KU170688	N/A
Dasella Lebour, 1945								
D. herdmaniae (Lebour, 1938)	IWP	Sabah, Malaysia	Tunicata: Ascidiacea: *Herdmania momus*	RMNH.CRUS.D.53924	N/A	KU064966	N/A	N/A
D. herdmaniae (Lebour, 1938)	IWP	Bali, Indonesia	Tunicata: Ascidiacea: *Herdmania momus*	RMNH.CRUS.D.49846	N/A	N/A	KU170689	N/A
Diapontonia Bruce, 1986								
D. maranulus Bruce, 1986	WA	Curaçao, West Indies	Echinodermata: Echinoidea: *Paleopneustes tholoformis*	RMNH.CRUS.D.57052	KU065050	KU064967	KU064815	N/A
Exoclimenella Bruce, 1995								
E. maldivensis Ďuriš & Bruce, 1995	IWP	Lizard Island, Australia	coral rubble	MTQ W-33394	KU065051	KU064968	KU064816	KU064886
E. sibogae (Holthuis, 1952)	IWP	Lizard Island, Australia	coral rubble	MTQ W-33263	KU065052	KU064969	N/A	KU064887
E. sudanensis Ďuriš & Bruce, 1995	IWP	Aqaba, Jordan	free-living	UO Aq09-6A	KU065053	KU064970	N/A	KU064888
Hamodactylus Holthuis, 1952								
H. aqabai Bruce & Svoboda, 1983	IWP	Aqaba, Jordan	Cnidaria: Alcyonacea: *Dendronephthya* sp.	UO Aq09-55	KU065054	KR088750	KU064817	KU064889
H. noumeae Bruce, 1970	IWP	Lizard Island, Australia	Cnidaria: Alcyonacea: *Isis* sp.	MTQ W-33246	KU065055	KU064971	N/A	KU064890
Hamopontonia Bruce, 1970								
H. corallicola Bruce, 1970	IWP	Lizard Island, Australia	Cnidaria: Scleractinia: *Goniopora* sp.	MTQ W-33307	KU065056	KU064972	KU064818	KU064891

H. fungicola Marin, 2012	IWP	Lizard Island, Australia	Cnidaria: Scleractinia: *Heliofungia actiniformis*	MTQ W-33109	KU065057	KU064973	KU064819	KU064892
H. physogyra Marin, 2012	IWP	Lizard Island, Australia	Cnidaria: Scleractinia: *Plerogyra* sp.	MTQ W-33313	KU065058	KU064974	KU064820	KU064893
Harpiliopsis Borradaile, 1917								
H. depressa Stimpson, 1860	IWP	Aqaba, Jordan	Cnidaria: Scleractinia: *Stylophora* sp.	UO Aq09-86	KU065059	KU064975	KU064821	KU064894
Harpilius Dana, 1852								
H. lutescens Dana, 1852	IWP	Aqaba, Jordan	Cnidaria: Scleractinia: *Acropora* sp.	UO Aq09-77	KU065060	KU064976	KU064822	KU064895
Ischnopontonia Bruce, 1966								
I. lophos (Barnard, 1962)	IWP	Lizard Island, Australia	Cnidaria: Scleractinia: *Galaxea fascicularis*	MTQ W-33360	KU065062	KU064978	KU064824	KU064897
Laomenes Clark, 1919								
L. amboinensis (De Man, 1888)	IWP	Taiwan	Echinodermata: Crinoidea*	UO Tw12-49	KU065063	KU064979	KU064825	KU064898
Lipkemenes Bruce & Okuno, 2010								
L. lanipes (Kemp, 1922)	IWP	Madang Bay, PNG	Echinodermata: Ophiuroidea*	MNHN IU-2013-10012	KU065064	KU064980	N/A	KU064899
Manipontonia Bruce, Okuno & Li, 2005								
M. psamathe (De Man, 1902)	IWP	Lizard Island, Australia	Cnidaria: Antipatharia*	MTQ W-33374	KU065066	KU064981	N/A	KU064900
Manipontonia sp.	IWP		Cnidaria: Antipatharia*	UO V08-43A	N/A	N/A	KU064826	KU170700
Nippontonia Bruce & Bauer, 1997								
Nippontonia sp.	IWP	Madang Bay, PNG	Cnidaria: Scleractinia: *Galaxea fascicularis*	MNHN IU-2013-10993	KU065067	KU064982	KU064828	KU064902
Odontonia Fransen, 2002								
O. sibogae (Bruce, 1972)	IWP	Aqaba, Jordan	Tunicata: Ascidiacea: cf. *Herdmania momus*	UO Aq08-T2	KU065068	KU064983	N/A	KU064903
Orthopontonia Bruce, 1982								
O. ornata (Bruce, 1970)	IWP	Madang Bay, PNG	Porifera: Demospongiae*	MNHN IU-2013-11102	KU065069	N/A	KU064829	KU064904
Palaemonella Dana, 1852								
P. rotumana (Borradaile, 1898)	IWP	Lizard Island, Australia	Cnidaria: Scleractinia: *Acropora* sp.	MTQ W-33176	KU065070	KR088755	KU064830	KU064905
Paranchistus Holthuis, 1952								
P. pycnodontae Bruce, 1978	IWP	Lizard Island, Australia	Mollusca: Bivalvia*	MTQ W-33124	KU065071	KU064985	KU064831	KU064906
Periclimenaeus Borradaile, 1915								
P. bidentatus Bruce, 1970	IWP	Lizard Island, Australia	Porifera: Demospongiae: *Acanthella cavernosa*	MTQ W-33404	KU065072	KU064989	KU064835	KU064909
P. pachydentatus Bruce, 1969	IWP	Bali, Indonesia	Tunicata: Ascidiacea: *Hypodistoma deerratum*	RMNH.CRUS.D.50157	N/A	N/A	KU170691	N/A
P. storchi Bruce, 1989	IWP	Sulawesi, Indonesia	Tunicata: Ascidiacea: *Didemnum* sp.	RMNH.CRUS.D.50154	N/A	N/A	KU064849	N/A
P. storchi Bruce, 1989	IWP	Sulawesi, Indonesia	Tunicata: Ascidiacea: *Didemnum* sp.	RMNH.CRUS.D.57053	KU065073	KU065008	N/A	N/A
Periclimenella Bruce, 1995								
P. spinifera (De Man, 1902)	IWP	Nhatrang Bay, Vietnam	coral rubble	UO V10-15A	KU065074	KU065007	KU064848	KU064922
Periclimenes G.O. Costa, 1844								
P. aegylios Grippa & d'Udekem d'Acoz,1996	EA	Brac Island, Crotia	Cnidaria: Actiniaria: *Anemonia* sp.	UO HR09-01	KU065075	KU064986	KU064832	KU064907
P. amethysteus (Risso, 1827)	EA	Pag Island, Crotia	Cnidaria: Actiniaria: *Anemonia* sp.	UO HR10-02	KU065076	KU064987	KU064833	KU064908
P. antipathophilus Spotte, Heard & Bubucis,1994	WA	Curaçao, West Indies	Cnidaria: Antipatharia*	RMNH.CRUS.D.57054	KU065077	KU064988	KU064834	N/A
P. brevicarpalis (Schenkel, 1902)	IWP	Nhatrang Bay, Vietnam	Cnidaria: Actiniaria: *Stichodactyla haddoni*	UO V08-22	KU065078	KU064990	KU064836	KU064910
P. colemani Bruce, 1975	IWP	Nhatrang Bay, Vietnam	Echinodermata: Echinoidea: *Toxopneustes* sp.	UO V08-104	KU065079	KU064991	N/A	KU064911
P. colesi De Grave & Anker, 2009	WA	Curaçao, West Indies	Porifera: *Callyspongia (Cladochalina) vaginalis*	RMNH.CRUS.D.57055	KU065080	KU064992	KU064837	N/A
P. commensalis Borradaile, 1915	IWP	Taiwan	Echinodermata: Crinoidea*	UO Tw12-48B	KU065081	KU064993	KU170697	KU064912
P. crinoidalis Chace, 1969	WA	Curaçao, West Indies	Echinodermata: Crinoidea: *Davidaster rubiginosus*	RMNH.CRUS.D.57056	KU065083	KU064995	KU064839	N/A
P. cristimanus Bruce, 1965	IWP	Nhatrang Bay, Vietnam	Echinodermata: Echinoidea: *Diadema* sp.	UO V08-34	KU065082	KU064994	KU064838	KU064913
P. granulimanus Bruce, 1978	IWP	Madang Bay, PNG	Cnidaria: Hydrozoa*	MNHN IU-2013-11097	KU065084	KU064996	KU064840	KU064914
P. inornatus Kemp, 1922	IWP	Lizard Island, Australia	Cnidaria: Actiniaria: *Stichodactyla* sp.	MTQ W-33160	KU065085	KU064997	KU064841	KU064915
P. kallisto Bruce, 2008	IWP	Lizard Island, Australia	Cnidaria: Antipatharia*	MTQ W-33346	KU065086	KU064998	N/A	KU064916
P. kempi Bruce, 1969	IWP	Lizard Island, Australia	Cnidaria: Alcyonacea*	MTQ W-33147	KU065087	KU064999	KU170695	KU064917
P. laevimanus Ďuriš, 2010	IWP	Nhatrang Bay, Vietnam	Cnidaria: Hydrozoa: cf. *Lytocarpia*	UO V08-118A	KU065088	KU065000	KU064842	KU064918
P. mclellandi Heard & Spotte, 1997	WA	Curaçao, West Indies	Cnidaria: Antipatharia*	RMNH.CRUS.D.51650	N/A	KU170687	KU170690	N/A
P. ornatus Bruce, 1969	IWP	Nhatrang Bay, Vietnam	Cnidaria: Actiniaria: *Heteractis* sp.	UO V08-80	KU065089	KU065001	KU064843	KU064919
P. patae Heard & Spotte, 1991	WA	Curaçao, West Indies	Cnidaria: Antipatharia*	RMNH.CRUS.D.57057	KU065090	KU065002	KU064844	N/A
P. perryae Chace, 1942	WA	Curaçao, West Indies	Echinodermata: Ophiuroidea: *Astrophyton muricatum*	RMNH.CRUS.D.57058	KU065091	KU065004	KU064845	N/A
P. rathbunae Schmitt, 1924	WA	St. Thomas, USVI	Cnidaria: Actiniaria: *Bartholomea annulata*	UO USVI-1	KU065093	KU065005	KU064846	KU064920
P. scriptus (Risso, 1822)	EA	Brac Island, Crotia	Cnidaria: Actiniaria: *Anemonia viridis*	UO HR09-03	KU065092	KU065006	KU064847	KU064921
P. wirtzi d'Udekem d'Acoz, 1996	EA	Madeira	Cnidaria: Antipatharia*	UO Md13-22	KU065094	KU065009	KU064850	KU064923
P. yucatanicus (Ives, 1891)	WA	St. Thomas, USVI	Cnidaria: Actiniaria: *Bartholomea annulata*	UO USVI-4	KU065095	KU065010	KU064851	KU064924
P. zanzibaricus Bruce, 1967	IWP	Taiwan	Echinodermata: Echinoidea: *Diadema* sp.	UO Tw12-86	KU065096	KU065011	KU170696	KU064925
Phycomenes Bruce, 2008								
Phycomenes sp.	IWP	Madang Bay, PNG	Cnidaria: Pennatulacea: *Virgularia* sp.	MNHN IU-2013-11046	KU065097	KU065012	KU064852	KU064926
P. sulcatus (Ďuriš, Horká & Marin, 2008)	IWP	Nhatrang Bay, Vietnam	free-living	UO V08-38	KU065098	KU065013	KU064853	KU064927
Platycaris Holthuis, 1952								

P. latirostris Holthuis, 1952	IWP	Aqaba, Jordan	Cnidaria: Scleractinia: *Galaxea* sp.	UO Aq09-45C	KU065099	KU065014	KU064854	KU064928
Pliopontonia Bruce, 1973								
P. furtiva Bruce, 1973	IWP	Nhatrang Bay, Vietnam	Cnidaria: Corallimorpharia: *Discosoma* sp.	UO V08-20	KU065100	KU065015	KU064855	KU064929
Pontonia Latreille, 1829								
P. pinnophylax (Otto, 1821)	EA	Cape Verde Islands	Mollusca: Bivalvia: *Pinna* sp.	RMNH.CRUS.D.42607	N/A	N/A	KU170692	N/A
Pontoniopsis Borradaile, 1915								
P. comanthi Borradaile, 1915	IWP	Taiwan	Echinodermata: Crinoidea*	UO Tw12-1	KU065101	KU065016	KU064856	KU064930
Rapipontonia Marin, 2007								
R. galene (Holthuis, 1952)	IWP	Lizard Island, Australia	Cnidaria: Hydrozoa: *Macrorhynchia philippina*	MTQ W-33104	KU065102	N/A	KU064857	KU064931
Stegopontonia Nobili, 1906								
S. commensalis Nobili, 1906	IWP	Nhatrang Bay, Vietnam	Echinodermata: Echinoidea*	UO V10-47	KU065103	KU065017	KU064858	KU064932
Thaumastocaris Kemp, 1922								
T. streptopus Kemp, 1922	IWP	Aqaba, Jordan	Porifera: Demospongiae: *Callyspongia* sp.	UO Aq09-15A	KU065105	KU065018	KU064860	KU064934
Tuleariocaris Hipeau-Jacquotte, 1965								
Tuleariocaris sp.	IWP	Aqaba, Jordan	Echinodermata: Echinoidea: *Echinothrix diadema*	UO Aq09-14	KU065106	N/A	KU064861	KU064935
Typton Costa, 1844								
T. wasini Bruce, 1977	IWP	Lizard Island, Australia	Porifera: Demospongiae: *Callyspongia* sp.	MTQ W-33302	KU065107	KU065019	KU064862	KU064936
Unguicaris Marin & Chan, 2006								
Unguicaris sp.	IWP	Taiwan	Echinodermata: Crinoidea*	NTOU 6687-09	KU065108	KU065020	KU064863	KU064937
Vir Holthuis, 1952								
V. euphyllius Marin & Anker, 2005	IWP	Nhatrang Bay, Vietnam	Cnidaria: Scleractinia: *Euphyllia* sp.	UO V10-46	KU065109	KU065021	KU064864	KU064938
V. orientalis (Dana, 1852)	IWP	Lizard Island, Australia	Cnidaria: Scleractinia: *Pocillopora* sp.	MTQ W-33130	KU065110	KU065022	KU064865	KU064939
V. philippinensis Bruce & Svoboda, 1984	IWP	Nhatrang Bay, Vietnam	Cnidaria: Scleractinia: *Plerogyra* sp.	UO V10-48	KU065111	KU065023	KU064866	KU064940
Zenopontonia Bruce, 1975								
Z. rex (Bruce, 1967)	IWP	Nhatrang Bay, Vietnam	Echinodermata: Holothuroidea: *Holothuria* sp.	UO V08-105	KU065112	KU065024	KU064867	KU064941
Z. soror Nobili, 1904	IWP	Nhatrang Bay, Vietnam	Echinodermata: Asteroidea: *Culcita* sp.	UO V08-111	KU065113	KU065025	KU064868	KU064942
**PANDALIDAE** Haworth, 1825 **(outgroup)**								
Miropandalus hardingi Bruce, 1983	IWP	Taiwan	Cnidaria: Antipatharia*	UO Tw11-20A	KU065065	KJ690259	KU064827	KU064901
Chlorotocella gracilis Balss, 1914	IWP	Nhatrang Bay, Vietnam	Porifera: Demospongiae: *Callyspongia* sp.	UO V10-42	KU065061	KU064977	KU064823	KU064896
**STENOPODIDAE** Claus, 1872 **(outgroup)**								
Stenopus hispidus (Olivier, 1811)	IWP	Nhatrang Bay, Vietnam	free-living	UO V10-17	KU065104	KJ690260	KU064859	KU064933

Used abbreviations and symbols: EA, East Atlantic; WA, West Atlantic; IWP, Indo-West Pacific; MNHN, National Museum of Natural History, France; MTQ, Museum of Tropical Queensland, Australia; NTOU, National Taiwan Ocean University; PNG, Papua New Guinea; RMNH, Naturalis Biodiversity Center, Leiden, the Netherlands; UO, University of Ostrava, Czech Republic; and USVI, U.S. Virgin Island; N/A - sequence not available; * - specific host not identified.

Supplementary Table S2: Additional sequences obtained from GenBank and used in the present analyses.

	GenBank accession numbers			
**Analysed taxa**	**H3**	**16S**	**18S**	**Source**
Anchiopontonia Bruce, 1992				
A. hurii (Holthuis, 1981)	N/A	KF738358	N/A	2
Araiopontonia Fujino & Miyake, 1970				
A. odontorhyncha Fujino & Miyake, 1970	N/A	KJ019633	N/A	3
Bathymenes Kou, Li & Bruce, 2015				
B. ngi (Li, Mitsuhashi & Chan, 2008)	N/A	JX025182	N/A	4
Cuapetes Bruce, 2004				
C. americanus (Kingsley, 1878)	N/A	EU868701	EU868795	1
C. elegans (Paul'son, 1875)	KJ584135	JX025213	N/A	4, 6
Dactylonia Fransen, 2002				
D. ascidicola (Borradaile, 1898)	KJ584137	N/A	N/A	6
Echinopericlimenes Marin & Chan, 2014				
E. dentidactylus Bruce, 1984	N/A	JX025189	N/A	4
E. hertwigi (Balss, 1913)	KJ019730	JX025186	N/A	4, 5
Gnathophyllum Latreille, 1819				
G. americanum Guérin-Méneville, 1855	KC515075	EU868660	EU868751	1, 5
Harpiliopsis Borradaile, 1917				
H. spinigera (Ortmann, 1890)	KF738319	JX025206	N/A	2, 4
H. beaupresii (Audouin, 1926)	KJ019704	JX025207	N/A	3, 4
Hymenocera Latreille, 1819				
H. picta	JF346328	EU868663	EU868754	1, 7
Laomenes Clark, 1919				
L. ceratophthalmus (Borradaile, 1915b)	KJ019709	JX025203	N/A	3, 4
Palaemonella Dana, 1852				
P. pottsi (Borradaile, 1915)	KF738324	JX025198	N/A	2, 4
Periclimenes G.O. Costa, 1844				
P. affinis (Zehntner, 1894)	KJ019725	JX025193	N/A	3, 4
P. boucheti Li, Mitsuhashi & Chan, 2008	KJ019727	JX025192	N/A	3, 4
P. laccadivensis (Alcock & Anderson, 1894)	KJ019732	JX025184	N/A	3, 4
P. leptunguis Li, Mitsuhashi & Chan, 2008	N/A	JX025183	N/A	4
P. sandybrucei Mitsuhashi & Chan, 2009	N/A	JX025179	N/A	4
Philarius Holthuis, 1952				
P. gerlachei (Nobili, 1905)	KJ019737	JX025177	N/A	3, 4
P. imperialis (Kubo, 1940)	KF738334	JX025176	N/A	3, 4
P. minor Marin & Anker, 2011	N/A	JX025175	N/A	4
Phycomenes Bruce, 2008				
P. cobourgi (Bruce & Coombes, 1995)	KJ019738	JX025174	N/A	3, 4

Sources:

1. Bracken, H. D., De Grave, S. & Felder, D. L. Phylogeny of the infraorder Caridea based on mitochondrial and nuclear genes (Crustacea: Decapoda). *Crustacean Issues* 18, 274-300 (2009).

2. Gan, Z., Li, X., Kou, Q., Chan, T.-Y., Chu, K. H. & Huang, H. Systematic status of the caridean families Gnathophyllidae Dana and Hymenoceridae Ortmann (Crustacea: Decapoda): a further examination based on molecular and morphological data. *Chin. J. Oceanol. Limnol.* 33, 149-158 (2014).

3. Gan, Z., Li, X., Kou, Q., Chan, Chu, K.H., & Kou, Q. Phylogeny of Indo-West Pacific pontoniine shrimps (Crustacea: Decapoda: Caridea) based on multilocus analysis. *J. Zoolog. Syst. Evol. Res.* 53, 282-290 (2015).

4. Kou, Q., Li, X. Z., Chan, T.-Y., Chu, K. H., Huang, H. & Gan, Z. B. Phylogenetic relationships among genera of the *Periclimenes* complex (Crustacea: Decapoda: Pontoniinae) based on mitochondrial and nuclear DNA. *Mol. Phylogenet. Evol.* 68, 14-22 (2013).

5. Kou, Q., Li, X. Z., Chan, T.-Y., Chu, K. H. & Gan, Z. B., Molecular phylogeny of the superfamily Palaemonoidea (Crustacea: Decapoda: Caridea) based on mitochondrial and nuclear DNA reveals discrepancies with the current classification. *Invertebr. Syst.* 27, 502-514 (2013).

6. Kou, Q., Li, X. Z., Chan, T.-Y. & Chu, K. H. Divergent evolutionary pathways and host shifts among the commensal pontoniine shrimps: a preliminary analysis based on selected Indo-Pacific species. *Org. Divers. Evol.* 15, 369-377 (2015).

7. Li, C. P., De Grave, S., Lei, H. C., Chan, T.-Y., Chu, K. H., Molecular systematics of caridean shrimps based on five nuclear genes: Implications for superfamily classification. *Zool. Anz.* 250, 270-279 (2011).

Supplementary Table S3: Alignment length (saturation test for protein-coding genes and GBlock for rRNA genes was applied), character variation, parsimony-informative sites, sample size and evolutionary models used for the genes in this study. Models for protein-coding genes (H3 and COI) are shown for the 1st, 2nd or 3rd codon positions. Used abbreviations: VS – variable sites, PI – parsimony-informative sites, N – number of sequences obtained within this study (with number of sequences used from GenBank in parentheses).

Four-gene analysis (90 taxa)

**Gene**	**Length** (bp)	**VS**	**PI**	**N**	**Models selected by PartitionFinder**
**H3**	293	105	94	89 (2)	GTR+I+Γ, JC+I, GTR+I+Γ
**COI**	436	121	89	85	SYM+I+Γ, GTR+I+Γ
**16S**	467	289	252	80 (5)	GTR+I+ Γ
**18S**	663	166	96	79 (3)	SYM+I+ Γ
**Total**	1859	681	531	333 (8)	

Three-gene analysis (112 taxa)

**Gene**	**Length** (bp)	**VS**	**PI**	**N**	**Models selected by PartitionFinder**
**H3**	293	105	93	102 (15)	GTR+Γ, JC+I, GTR+I+Γ
**COI**	436	121	89	85	SYM+I+Γ, GTR+I+Γ
**16S**	478	307	260	105 (22)	GTR+I+ Γ
**Total**	1207	533	442	292 (37)	

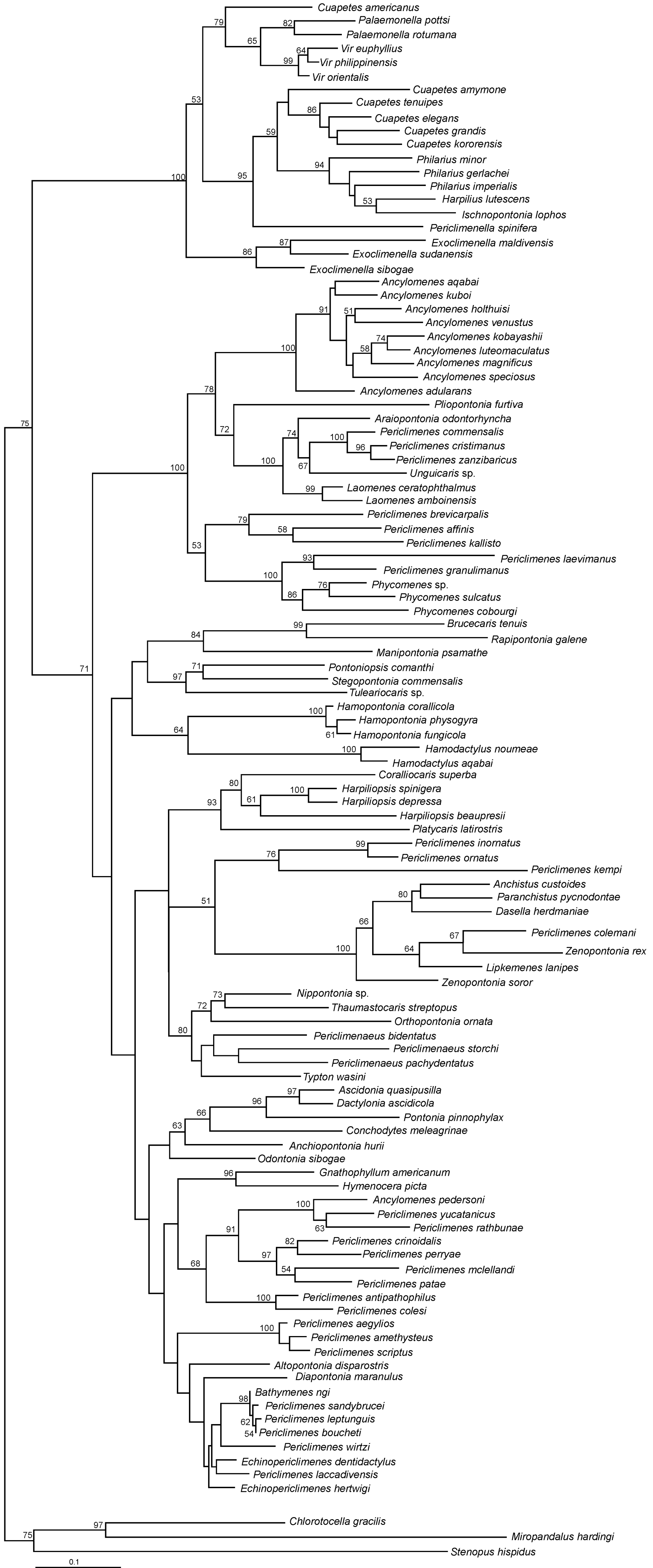

Supplementary Figure S1.

Phylogenetic tree of symbiotic Palaemonidae shrimp taxa (including sequences of taxa retrieved from GenBank) resolved by RAxML analysis based on the combined dataset for three genes (COI, 16S, H3). Bootstrap supports >50 are displayed.
